# SBRT for Localized Prostate Cancer: CyberKnife vs. VMAT-FFF, a Dosimetric Study

**DOI:** 10.3390/life12050711

**Published:** 2022-05-10

**Authors:** Marcello Serra, Fortuna De Martino, Federica Savino, Valentina d’Alesio, Cecilia Arrichiello, Maria Quarto, Filomena Loffredo, Rossella Di Franco, Valentina Borzillo, Matteo Muto, Gianluca Ametrano, Paolo Muto

**Affiliations:** 1 Istituto Nazionale Tumori—IRCCS—Fondazione G. Pascale, 80131 Napoli, Italy; valentina.dalesio@istitutotumori.na.it (V.d.); c.arrichiello@istitutotumori.na.it (C.A.); r.difranco@istitutotumori.na.it (R.D.F.); v.borzillo@istitutotumori.na.it (V.B.); gianluca.ametrano@istitutotumori.na.it (G.A.); p.muto@istitutotumori.na.it (P.M.); 2 Dipartimento di Scienze Biomediche Avanzate, Università degli Studi di Napoli Federico II, 80131 Napoli, Italy; fo.demartino@studenti.unina.it (F.D.M.); maria.quarto@unina.it (M.Q.); filomena.loffredo@unina.it (F.L.); 3 LB Business Services SRL, 00168 Rome, Italy; savino.federica@gmail.com; 4 Division of Radiotherapy, “S. G. Moscati” Hospital, 83100 Avellino, Italy; matteo.muto@aornmoscati.it

**Keywords:** SBRT, hypofractionation, CyberKnife, VMAT, flattening filter free, prostate cancer

## Abstract

In recent years, stereotactic body radiation therapy (SBRT) has gained popularity among clinical methods for the treatment of medium and low risk prostate cancer (PCa), mainly as an alternative to surgery. The hypo-fractionated regimen allows the administration of high doses of radiation in a small number of fractions; such a fractionation is possible by exploiting the different intrinsic prostate radiosensitivity compared with the surrounding healthy tissues. In addition, SBRT treatment guaranteed a better quality of life compared with surgery, avoiding risks, aftermaths, and possible complications. At present, most stereotactic prostate treatments are performed with the CyberKnife (CK) system, which is an accelerator exclusively dedicated for stereotaxis and it is not widely spread in every radiotherapy centre like a classic linear accelerator (LINAC). To be fair, a stereotactic treatment is achievable also by using a LINAC through Volumetric Modulated Arc Therapy (VMAT), but some precautions must be taken. The aim of this work is to carry out a dosimetric comparison between these two methodologies. In order to pursue such a goal, two groups of patients were selected at Instituto Nazionale Tumori—IRCCS Fondazione G. Pascale: the first group consisting of ten patients previously treated with a SBRT performed with CK; the second one was composed of ten patients who received a hypo-fractionated VMAT treatment and replanned in VMAT-SBRT flattening filter free mode (FFF). The two SBRT techniques were rescaled at the same target coverage and compared by normal tissue sparing, dose distribution parameters and delivery time. All organs at risk (OAR) constraints were achieved by both platforms. CK exhibits higher performances in terms of dose delivery; nevertheless, the general satisfying dosimetric results and the significantly shorter delivery time make VMAT-FFF an attractive and reasonable alternative SBRT technique for the treatment of localized prostate cancer.

## 1. Introduction

Prostate cancer (PCa) is a broad disease affecting male population; it is the second most frequent cancer diagnosed in men after the lung cancer, and it is the fifth cause of death in the world. To understand the extent of the problem, worldwide in 2018, about 1,300,000 new cases of PCa were reported (in Italy, about 36,000 new cases in 2020 [1]), and it caused about 7700 deaths [1,2]. Moreover, the risk to be diagnosed with PCa is strictly linked with aging, and the incidence rate varies across the world but it can be resumed as follows: in men under 39 years old, the probability is about 0.005%; between 40 and 59 years old, the probability rises to 2.2%; between 60 and 79 years old, the probability is 14%; and over 80 years old, the incidence is very high, at about 50% [3]. Given the high incidence of the illness and the lengthening of life expectancy, the population affected by this pathology is estimated to increase; indeed, about 2,300,000 new cases are expected up to 2040 [4]. Today, patients with localized disease and detected at early stage at low to intermediate risk of recurrence have a favorable prognosis: 99% overall survival for 10 years. As matter of fact, the localized PCa shows a high patient’s life expectancy, a slow progression rate, and limited metastatic potential; therefore, in this class of patient, comorbidities are considered very significant, since the increase of comorbidities with a poor health status, due to the aging, increase the risk of dying from other causes than PCa [5].

When it comes to the fight against cancer, everyone thinks about surgery [6]; however, for PCa, surgery is not the only way to go. Today, we have different therapeutic approaches, and the best choice is based on the tumor risk class, the patient performance status and, no less important, it depends on the patient’s preferences and assessment of side effects. Radiotherapy (RT) is especially effective for the PCa treatment due to the considerable difference of the α/β ratio between the tumor and the surrounding Organ at risk (OAR) [7,8,9] (bladder, rectum, penile bulb and bowel). Such a difference makes it possible to perform hypofractionated treatment. In 2018, international societies such as ASTRO, ASCO and AUA, after deep studies, reported that there are solid grounds to support the use of the hypofractionated regime for treatment of the PCa for the routine clinical practice [10], and they also recommend the Stereotactic body radiation therapy (SBRT) approach for clinical trials in patients with high risk localized PCa [11,12]. A systematic review of studies where a comparison among SBRT, hypofractionated and normofractionated regimes concluded that SBRT achieves the same results of the other treatment modalities in terms of five years disease free survival, but a reduced gastrointestinal and genitourinary toxicity, <15% and 21% respectively [11].

It is very interesting to investigate if rotational approaches, such as VMAT Volumetric modulated arc therapy (VMAT), can potentially deliver a SBRT treatment as good as that of CyberKnife (CK). The aim of this study is to investigate, retrospectively, the use VMAT with 6-FFF MV for the PCa treatments, and to analyse the differences with the CK system, already adopted in our department. The comparison of these two SBRT will be carried out in terms of tumor coverage and OARs spearing, through relative dosimetric parameters, by analysing and comparing plans and the respective Dose volume histogram (DVH) curves, for the purpose of expanding the use of the SBRT as much as possible, even in radiotherapy centres where a CK system is not available.

## 2. Materials and Methods

### 2.1. The CyberKnife System

In RT, the platform specifically designed for the SBRT is the CyberKnife (CK, Accuray Inc., Sunnyvale, CA, USA), and indeed due to its peculiarities, promising outcomes have been achieved. These results are confirmed by 8- to 10- year studies, which support the clinical evidence of the successful employment of SBRT for localized PCa [13,14,15]. The CK is a linear accelerator of 6 MeV energy installed on a robotic arm with six degrees of freedom; therefore, it is characterized by a non-isocentric dose delivery mode. Moreover, it also ships static circular collimators from 5 to 60 mm, or a dynamic IRIS collimator. In particular, the accelerator can be positioned in 100 nodes and, in each of them, it can take up to a maximum of twelve directions: in that way, 1200 different entry beam directions can be reached. By exploiting its considerable mobility and adaptability of the radiation field, high dose conformation level is achieved; furthermore, CK can deliver the 125% of the prescribed dose in the tumor volume, but at the same time achieving significant OARs sparing.

CK must deliver the dose with surgical precision, otherwise undesired volumes and organs can be reached by high doses and the lesion can be severely underdosed; therefore, CK is equipped with an image-guided system, useful both for the correct positioning of the patient and to monitor the movements of the target during the treatment. The image system is composed by two X-ray tubes stuck to the ceiling by 90° to each other and tilted with respect the patient’s axis by 45°; X-ray tubes are correlated to a pair of silicon detectors (flat panel) placed in the floor, next to the treatment bed. The nominal tube voltage is 40 keV–15 MeV. The patient is imaged every 45 s, or at most 60 s, and the live images are digitized and compared to images synthesized from the patient’s CT data (digitally reconstructed radiograph (DRR)). This technique allows for determination of intra-fraction target shifts and automatic compensation by the treatment manipulator during treatment delivery. These automatic corrections are achievable for a maximum excursion range: X, Y and Z direction ± 10 mm, pitch, roll and yaw ±5°, ±1°, ±3°, respectively. For shifts greater than these intervals, the treatment is interrupted, and an operator repositions the patient [16].

In order to locate the tumor, in our case the prostate, and to check if its position changes during the treatment, some golden fiducials (usually four) are implanted in the prostate wall; the reconstruction of the prostate position by means of such markers allows adjustments of the accelerator real-time with respect to the target [17,18,19]. For the sake of completeness, in RT, when an accelerator is supported by an image system, the technology is called image guided radiotherapy (IGRT), and the use of accurate image guidance is crucial to minimize setup errors and facilitate the margins reduction between the gross tumor volume (GTV) and the Planning target volume (PTV), especially for SBRT.

### 2.2. SBRT-VMAT and FFF Delivery Mode

A SBRT treatment is also achievable by using a state-of-the-art LINAC guided by a software conceived for the VMAT. VMAT combines the intensity modulation of the radiant field with its shape adaptability through a multi-lamellar collimator (MLC). The dose is delivered continuously during the gantry rotation along one or more arcs without interruptions and, over the treatment, the dose rate varies as well. These features allow a dose distribution to be reached that is extremely compliant to the target volume with a greater sparing of normal tissue than static 3D-CRT. 

Due to the advent of systems for positioning verification and the movement monitoring of organs and anatomical volumes of interest, it is possible to achieve better safety in the administration of ultra-hypo-fractionated regimens, and therefore it is possible to develop real stereotaxic treatments also with the VMAT technique.

The LINAC used in this work is the Elekta Versa HD; it has photon energy of 6, 10, and 15 MeV, but for the VMAT treatment, just 6 MeV energy is enabled. The maximum field size for a treatment is (40 × 40) cm^2^, defined by a pair of fixed collimators, that can rotate independently of the gantry, mounted orthogonally to the MLC. The MLC consists of 80 pairs of tungsten blades of a projected width of 5 mm at the isocentre, and a small interleaf space less than 0.1 mm. As noted above, in order to perform a SBRT treatment, an IGRT system is required; indeed, in our case, the accelerator is equipped with an ultrasound guidance system released for VMAT treatment: the Clarity System [20] (Elekta, Stockholm, Sweden). It consists of a transperineal ultrasound (not ionizing radiation) probe, which allows real-time prostate visualization during the treatment, and which can stop if the prostate makes too large excursions with respect to the planning Computed tomography (CT). The Clarity System reconstructs a 3D image starting from 2D ultrasound acquisitions. To obtain images, the probe needs to be moved, but during the treatment the radiotherapist is not in the treatment room; therefore, the probe performs automatic scans with a motorized control of the sweeping motion. The probe can scan a complete 75° sweep in 0.5 s. Patients do not feel any discomfort other than a slight vibration, as all motion is internal to the probe housing. Moreover, the probe has an integrated sensor which triggers when it passes through the center. Every sweep is checked for geometrical accuracy [21]. Therefore, such a probe allows a more efficient verification of the position of the tumor and critical structures; indeed, the employment of this technique shows a significant reduction in matching errors compared to standard treatments [20,21]. We want to highlight that this continuous monitoring does not imply a higher dose administration to the patient, since we are dealing with ultrasound images and no additional device needs to be implanted in the patient’s prostate [22].

In order to compute and deliver a therapeutic plan which reaches inside the tumor volume, a dose higher than the prescribed dose with a very steep dose gradient, the accelerator must operate in FFF mode; that is, in the absence of the homogenizer filter. The beam emerging from the primary collimator is extremely spiked, due to the greater probability of production of bremsstrahlung photons for small angles with respect to the direction of motion of the incident electron. The result is a non-homogeneous beam, with a maximum of intensity in the centre and a decreasing intensity at the sides. The use of the filter reshapes the beam profile, attenuating it in its central region and widening it by diffusion, thus giving it an almost flat structure. The beam is also hardened since the filter removes most of the low-energy photons by attenuation. On the contrary, when operating in FFF mode, the radiation beam appears narrower and more intense in the central part, which is useful to better conform the dose distribution to the target. Furthermore, the presence of the soft component of the beam leads to an increase in the dose-rate and the consequent reduction in treatment delivery times. This situation helps to minimize the effects of movement of the target and is particularly advantageous in stereotaxic therapies where extremely high doses are delivered and accuracy in the delivery of treatment is of crucial importance.

In general, the beam-on time tends to be longer in SBRT, since higher doses per fraction are prescribed; as a result, the prostate position can change during the treatment, due to rectal activity, bladder filling, muscle clenching and general pelvic motion. As matter of fact, while setting and performing RT, it is important to be able to predict the occurrence and the extent of the prostate movement to establish appropriate planning target volume (PTV) margins, avoiding a missing target. Nevertheless, FFF beams, which have higher dose rates, decrease the treatment time per fraction; therefore, the faster delivery makes the treatment patient-friendly and improves treatment accuracy by preserving the treatment plan quality [23,24], by reducing the intra-fractional motion.

One of the most important aspects of the SBRT is the conical dose distribution with a maximum value that can reach the 125% of the prescribed dose, located near the geometric centre of the target, and a sudden decrease outside the target. Traditional radiotherapy treatments, on the other hand, require a uniform dose on the target, that cannot exceed the 110% of the prescribed dose; small hot spots can sometimes be accepted. From these conceptual differences, it can be derived that the typical dose–volume histogram (DVH) structure of a traditional VMAT treatment is very different to a stereotaxic one. Indeed, in the first case, DVH looks like a step function extending from 0 Gy to the prescription dose value. In a stereotaxic plane, the decreasing part of the graph has a gentler slope, and the maximum dose can reach 125% of the prescribed dose. Such high dose values at the target have precise clinical implications, as they could offer a special advantage in the eradication of radio-resistant hypoxic cells.

**Figure 1:**
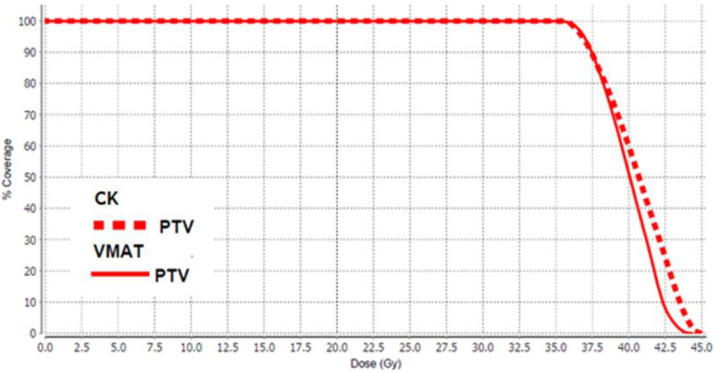
The Planning target volume (PTV) Dose volume histogram (DVH) for the CyberKnife (CK) (dotted line) and Volumetric modulated arc therapy (VMAT)-Flattening Filter Free (FFF) (solid line) techniques. With the specific measures adopted in the planning phase, the VMAT curve looks very similar to the CK one.

To obtain the typical SBRT dose distribution with the VMAT treatment modality, some tricks had to be adopted in the planning process. For each patient, an auxiliary fictitious structure was delineated at the centre of PTV, and the parameters of suited cost functions were given appropriate values. In the inverse planning, these dodges have forced the optimization algorithm to reshape the dose distribution in order to resemble the typical DVH appearance of a stereotaxis treatment (Figure 1).

### 2.3. The Dosimetric Study: Patient Selection, Contouring and Planning

Since the two platforms involved in the study have an inherently different way of delivering the dose, and a different IGRT system which can affect the anatomical configuration of the organs, to have a truly representative dosimetric study, we compared patients who were imaged and have received CK treatment with patients who were imaged and received treatment with the VMAT by using the Clarity system. Of course, for the VMAT patients, the therapeutic plans were re-computed in accordance with the ultra hypofractionated prescription: 36.25 Gy in five fractions. At this point, it could be argued that the best or worst results achieved with CK or with SBRT-VMAT would not be directly comparable since they could depend on specific patient anatomical characteristics. Therefore, in order to reduce and smooth such patient’s differences, groups of patients were selected, and dosimetric results are expressed as average values. 

For sake of completeness, in our institute as actual clinical practice, the patients selected for SBRT with CK satisfy the following settings: age > 18 years; PCa diagnosed with transperineal core biopsy; low risk class (T2a or lower, Prostate Specific Antigen (PSA) ≤ 10 ng/mL and Gleason Grade (GG) = 1); intermediate risk class (T2b-2c, PSA ≤ 20 ng/mL and GG = 2); prostate volume < 80 cc; uninvolved prostate capsule (documented by prostate multiparametric Magnetic Resonance Imaging (MRI)); performance status: 0–1; TC total body with contrast enhancement bone scintigraphy negative for metastasis (Table 1).

Two groups of patients with a localized PCa, each of them composed of 10 patients treated in Instituto Nazionale Tumori—IRCCS Fondazione G. Pascale in the period between 2016 and 2019, were selected. The first group (Table 1), that from here on it will be named the “CK Group”, consisting of ten patients of, on average, 69 years old and having an average prostatic volume of 66 cc, were treated with SBRT realized by the CK system with a dose prescription of 36.25 Gy to the PTV in five fractions. The second one, named the “Clarity Group” (Table 1), comprising of, on average, 76-year-old patients with an average prostatic volume of 44 cc. They were treated with a hypo fractionated VMAT delivered by an Elekta Versa HD matched with the Clarity ultrasound probe, with a dose prescription of 35 Gy to the PTV in five fractions. By taking advantage of the CT scans with the Clarity probe in position, they have been replanned with an optimized template developed for prostate VMAT-SBRT in FFF mode with the same dose prescription and fractionation scheduled for the CK Group.

Regarding volumes contouring, in both groups, the GTV was defined as prostate without margin, the CTV was set equal to GTV, and the PTV was CTV with 3 mm expansion posteriorly and 5 mm in other directions. The rectum, rectal wall, bladder, bladder wall, bowel, penis bulb and femoral heads were delineated and classified as OARs. The rectal and bladder walls were contoured through a Boolean subtraction between the organ itself and its own contractions, respectively, of 3 mm and 5 mm.

For the IGRT system of the CK Group, four gold fiducials markers have been implanted on prostate surface 7/10 days before CT images acquisition. For Clarity Group patients, the ultrasound probe Clarity was used as a volume monitoring device, and CT images were acquired with the probe in position, compressing perineal region.

The CK treatment planning was performed using the precision inverse treatment planning system (Accuray Inc., Sunnyvale, CA, USA), and the prescription dose was 36.25 Gy at 80% isodose line delivered in five fractions, with a beam energy of 6 MeV, with the dynamic IRIS collimator. The OARs constraints used for planning were V_37.5 Gy_ < 5 cm^3^ for the bladder and V_36.25 Gy_ < 5% for the rectum, and three concentric shells were used for dose conformation to PTV. VMAT-SBRT treatment plans were computed with Elekta Monaco TPS and the treatments geometrical setup was: 2 arcs of 360° (clockwise and counterclockwise) with a 10° collimator tilt angle and final gantry spacing 3°; in both arches, there was a 0° couch kick. VMAT plans were computed for an Elekta VERSA HD accelerator (Elekta, Crowley, UK) equipped with 6 MeV photon beam in FFF mode and MLC with 5 mm leaf.

In both cases, the dose distribution was renormalized as a 95% prescription dose, covering 95% of the PTV.

## 3. Statistical Analysis

The VMAT-FFF therapeutic plans were computed by experienced medical physicists, and after, DVHs curves were extracted and analysed with Microsoft Excel. In Table 2, the dose received by a specified volume percentage and the volume that received a certain amount of dose of the respective ROI are presented. Table 3 reports the mean duration of treatment and the parameters characterizing the dose distribution: the homogeneity indexes (HI), the conformality index (CI), and the gradient index (GI).

Usually, HI deals with the degree of uniformity of dose distribution within target; nevertheless, stereotaxic treatments, by definition, produce an inhomogeneous dose distribution in the target, so in the statistical analysis we referred to a homogeneity index definition that could quantify the dose peak height within the PTV.

For the evaluation of this parameter, we referred to the following equation:

(1) $$HI=\frac{D_{MAX}}{R_{XDose}}\;$$

D_MAX_ is the maximum dose value at the PTV, and R_XDose_ is the prescription dose value. Theoretically speaking, this value could be 1 in the case of perfect homogeneity, but in our case, we are aware that in an SBRT treatment it is expected to be higher than 1.

CI deals with the degree of compliance of the dose distribution with the target volume; it is defined as the ratio of total volume of tissue treated with a prescription dose over the volume of the tumor treated with the prescription dose:

(2) $$CI=\frac{PIV}{TIV}$$

PIV is the prescription isodose volume (total 3D volume of the isodose line), and TIV is the tumor isodose volume (tumor volume covered by the prescription isodose volume). From a mathematical point of view, CI should be 1 if the prescription dose volume covers the tumor volume perfectly; therefore, a CI closer to 1 is desirable. GI allows to quantify the steepness of the dose fall-off in a stereotaxic treatment. It is defined as the ratio between the volume of a reference isodose and the volume of the prescription isodose:

(3) $$HI=\frac{D_{MAX}}{R_{XDose}}$$

The lower the GI value, the more the considered isodose will adhere to that relating to 100% of the prescribed dose, and the steeper the dose falls outside the target.

## 4. Results

To give an overview about the treatments, the most relevant constraints used in ultra hypo-fractionated radiotherapy for PCa, and also the values reached by using the two aforementioned technologies, are shown. For OARs, the maximum dose and the most significant values extracted from the DVH curves, the CI, HI and GI indices, and the beam-on time have been reported. They are listed in Table 2 and Table 3. In general, from the data analysis emerges a substantial equivalence between the techniques; both of them comply with dose constraints and lead to comparable doses delivered to OARs.

The values in the table indicate that for the bladder: D_1cc_ is the dose absorbed by 1 cc of volume, V_37.5_ and V_37_ are the volumes receiving a dose of 37.5 Gy and 37 Gy, V_36.25_ and V_18.125_ are the volumes receiving the 100% or the 50% of prescription dose, whereas V_5_, V_10_ and V_20_ are the volumes receiving 5 Gy, 10 Gy and 20 Gy, respectively. For the rectum, the bladder wall, bowel, penis bulb, left femoral head (LFH) and right femoral head (RFH), listed in the Table 2 as well, different values of dose and volumes are reported, but the indices have the same significance exposed before for the bladder case.

Regarding the bladder, we can assert that both the maximum dose and the reference constraints values are comparable and consistent according to the two-tailed test. The same holds true for the volume of the isodose curves relative to the 20 Gy and 10 Gy dose values. The only significant difference is highlighted at low doses: the VMAT technique shows a greater bladder sparing, which can be displayed by the volume surrounded 5 Gy isoline. The bladder wall takes a comparable dose in the two techniques; instead, the discrepancy between dose values is not significant.

Regarding the rectum and the rectal wall, all values relating to dose and volume constraints are comparable and consistent according to the t-test. Additionally, for the bowel, VMAT shows better organ sparing at low doses, as evidenced by the V_10 Gy_ isosurface.

Regarding the penis bulb, in the VMAT technique, a larger dose is absorbed, and such a difference becomes more and more appreciable at low doses.

No substantial difference was highlighted for the femoral heads, which for both techniques are well below the dose constraints.

Finally, by looking at Table 3, regarding dose distribution descriptive parameters, a better dose conformation to PTV is evidenced for VMAT as demonstrated by CI values. As expected, the typical conical dose distribution of stereotaxis is more pronounced in the CK case, as can be deduced from HI values. About GI values, they are all comparable, and the discrepancies are not statistically significant (*p*-value > 0.05). From this, it can be deduced that the trend of the dose gradient outside the PTV is very similar in the two techniques. Regarding the duration of the treatment, the VMAT-FFF allows a much higher dose rate than the CK, enabling a shorter beam-on time.

## 5. Discussion

As evidenced by the epidemiologic data, PCa is a common disease related to aging with an increasing number of cases projected into the future. RT is carving out more and more space in the fight against the PCa due to its lower invasiveness with respect to surgery. In low risk PCa, exclusive radiotherapy and radical prostatectomy are equivalent and show similar clinical outcomes (overall survival and quality of life) [25]. In the RT field, the gold standard is 2 Gy per fraction, whereas hypofractionation enables higher doses to be reached in fewer sessions. In other words, the α/β for prostate is very low (about 1.5 Gy) if compared to the OARs; therefore, it allows the maximization of the therapeutic effect, and at the same time reduces side effects on OARs as much as possible [26,27,28,29], increasing the therapeutic window, i.e., the distance between the tumor control probability (TCP) curve and normal tissue complication probability (NTCP) curve [27]. The evolution of radiotherapy knowledge, and the fast technology improvement, that led to high precision treatments guided by real-time imaging, have allowed the consideration that the SBRT is a promising treatment for this disease. The SBRT is an extremization of the hypofractionated regime, with very few sessions and very high doses; indeed, nowadays, SBRT using a five-fraction schedule appears to be an effective treatment for low- and intermediate- risk PCa in terms of efficacy, safety, patient’s quality of life and side effects, as established by the literature [30,31,32,33]. As pointed out by other works, the SBRT in an ultra hypofractionated regime is very effective in terms of overall survival and quality of life; indeed, the recent HYPO-RT-PC study provided evidence that 42.7 Gy delivered in seven sessions every other day (6.1 Gy per fraction) has comparable outcomes to standard conventional fractionation of 78 Gy in 2 Gy per fraction [11]. That is, the same result is achieved in fewer sessions, resulting in less stress for the patient and reduced costs for the hospital. Other studies with long term follow up and a large cohort of patients recommend prescription doses between 35 Gy and 36.25 Gy in five fractions [34]. Some authors indicate that patients with low- and intermediate risk disease treated with 31.7 Gy in five fractions and 36.1 Gy in five fractions achieved tumor control probabilities of 90% and 95%, respectively, at five years follow up [35]. Nevertheless, a schedule of 40 Gy in five fractions has shown higher toxicity with respect to the previous radiotherapy programs [36]. In addition, if some works look at a short time window after the treatment time, such results corroborate the idea that the dose escalation could increase the effectiveness of the RT for the PCa, as long as an EQD2 < 100 Gy1.5 is delivered. Furthermore, studies are still in progress to validate the use of even higher doses for high-risk PCa.

SBRT treatment is typically delivered by using a CK, and in the current context its usage is increasing, but technological improvements allowed they delivery of SBRT treatments with isocentric technique, such as VMAT and helical tomotherapy (HT). There is a wide range of literature about comparisons among rotational techniques and CK bringing extremely encouraging outcomes and resulting in a general agreement among different data [37,38,39,40,41,42]. The SBRT delivered by VMAT is a viable option, and the main differences found in such works are due to the PTV coverage and the margins expansion from the CTV to the PTV. These two points are essential because rotational techniques are routinely used to reach a PTV homogeneous coverage (the perfect PTV-DVH curve could be a step function); however, in a CK-like SBRT, 125% of the prescription dose is deposited in the inner parts of the tumor. If in VMAT and HT no tumor tracking system is used, a wider PTV contouring should be drawn. Hence, even if the two techniques show comparable OARs, sparing the dose in tumor could be significatively lower in VMAT/HT cases with respect to CK ones, and such differences could have important radiobiological consequences; on the other hand, to reach the highest CK doses, if no unsuitable contouring is drawn, dangerous dose spilling is expected. Among rotational techniques for the SBRT, one of the latest developments is the MRI-Linac; it is a Linac equipped with a MRI scanner. Due to the peculiarity of the MRI images to contrast better soft tissues, such technology can deliver high doses with high accuracy. Results reported in the literature show that SBRT treatments supported by MRI images can achieve significant OARs sparing [43,44]. As for some works summarized before, a comparison with our results is not directly practicable, as in MRI-Linac based studies it is accepted that, at most, 107% of the prescription dose reaches the PTV; whereas, in our analysis, we are reaching doses higher than 120% in order to simulate a CyberKnife-like treatment. Nevertheless, even if in this work and MRI-Linac studies the selected constraints for the bladder and rectum are different (but similar), comparable results are reached between the exhibited techniques.

Therefore, to have a significant comparison among the VMAT and CK treatments, we computed VMAT plans in FFF mode in order to obtain the conical dose escalation contemplated by CK, and such plans were worked out on TC with the IGRT system in position so that the same geometrical rules for the PTV delineation could be applied in both cases.

From the results listed in Table 2, the most important discrepancies can be outlined for the bladder and bowel, at low doses; probably such differences could be due to the dose delivery modalities of the two techniques: the freedom of movement of the non-isocentric CK approach implies beam directions coming from a solid angle intercepting larger OARs volume before reaching the prostate with respect to a well delimited strip dose typical of rotational techniques. Such different dose structures may involve the production of low dose spikes that fall extensively within the bowel and bladder volume. It is worth noting that also in previous works there were similar dose structures, with the more interesting differences reached at the lowest or intermediate doses; this should further confirm the idea that the delivering dose modality could also play an important role in the acute and late toxicity.

The other clear disagreement can be found in the penis bulb dose distribution. It can be easily ascribed to the use of the Clarity probe for VMAT IGRT. During the treatment, the ultrasound probe is positioned close to the perineal region, compressing the penis bulb; this arrangement involves an anatomical deformation which brings the penis bulb closer to the prostate, and hence the probe determines a greater absorbed dose to the bulb.

At last, but not the least, the most important difference in the beam is time: VMAT treatments are much faster. This is very important because the time reduction results in greater patient comfort, the natural and uncontrollable organ movements are reduced during the treatment time, and as consequence, a clinical centre could dramatically shorten its waiting list.

We want to highlight that these results are based on small groups of patients (ten for each technique); therefore, to have a more robust statistical significance, the number of patients involved in the dosimetric study should be enlarged. Moreover, in this study, the prostate is not the exclusive target, but in some cases, the PTV is composed of prostate and seminal vesicles. For the sake of completeness, this mix of targets is present in both groups of patients (CK Group and Clarity Group). A further analysis, having at disposal a larger dataset, could be carried out by dividing the low risk PCa patients into two subgroups, in order to analyze if such subdivision can have repercussions on the performance of the two techniques. Finally, none of the patients involved in the study had comorbidities detectable in the TC images, such as diverticula, hernias, etc., which could have influenced the planning process.

## 6. Conclusions

The authors, with this publication, state that SBRT VMAT-FFF is a noteworthy technique which might substitute, in some cases, the CK. Our data show that VMAT-FFF with the Clarity system could be used for the hypofractionated treatment of PCa with good clinical results and while respecting the recommended constrains. Therefore, the ultra hypofractionated regime, and more generally the SBRT, with its important clinical and managerial implications, might be spread also in smaller radiotherapy centers where a CK is not available.

## Figures and Tables

**Table 1:** Summarized clinical and demographic characteristics of the population involved in this study.

Patient	Age	Total PSA	Target Volume (cc)	Gleason Score (GS)	Clinical Stage
1 (CK)	72	5.63	51.06	1	1c
2 (CK)	72	10.84	85.82	1	1c
3 (CK)	65	16.6	62.26	1	2b
4 (CK)	70	6.3	44.08	2	2a
5 (CK)	65	7.32	36.48	1	1b
6 (CK)	76	8.63	86.90	1	2a
7 (CK)	63	4	47.08	2	2a
8 (CK)	62	4.5	80.12	2	2b
9 (CK)	72	5.5	66.36	1	2a
10 (CK)	73	7.6	72.35	2	2a
1 (VMAT-FFF)	78	7.29	37.75	2	1c
2 (VMAT-FFF)	78	4.37	31.10	2	2b
3 (VMAT-FFF)	77	10.69	30.78	2	2b
4 (VMAT-FFF)	81	8.71	33	2	
5 (VMAT-FFF)	76	9.6	68.17	1	2b
6 (VMAT-FFF)	78	7.22	36.28	1	2b
7 (VMAT-FFF)	72	12	53.91	1	1c
8 (VMAT-FFF)	72	6.21	44.55	1	2b
9 (VMAT-FFF)	78	11	23.74	2	2b
10 (VMAT-FFF)	70	6.76	79.75	1	2a

**Table 2:** The table summarizes the most significant values of the DVH curves achieved by the two techniques.

	Parameter	CK	VMAT	*p*-Value
Bladder	D_MAX_ (Gy)	39 ± 2	41 ± 2	0.1
	D_1 CC_ (Gy)	37 ± 2	38 ± 1	0.05
	V_37.5 Gy_ < 5 cm^3^	1 ± 2	3 ± 2	0.1
	V_37 Gy_ < 10 cm^3^	2 ± 2	4 ± 2	0.2
	V_36.25 Gy_ < 10%	2 ± 2	3 ± 1	0.2
	V_18.125 Gy_ < 40%	37 ± 12	35 ± 10	0.7
	V_5 Gy_ (%)	92 ± 11	68 ± 22	0.02
	V_10 Gy_ (%)	76 ± 18	57 ± 21	0.06
	V_20 Gy_ (%)	32 ± 11	30 ± 9	0.7
Bladder wall	D_MAX_ (Gy)	40 ± 2	41 ± 1	0.3
	D_10 cm^3^_ (Gy)	32 ± 3	33 ± 2	0.5
Rectum	D_MAX_ Gy	37 ± 2	37 ± 1	0.3
	V_36.25 Gy_ < 5 cm^3^	0.2 ± 0.3	0.5 ± 0.7	0.1
	V_36 Gy_ < 1 cm^3^	0.1 ± 0.2	0.5 ± 0.6	0.07
	V_32.625 Gy_ < 10%	4 ± 2	4 ± 3	0.5
	V_29 Gy_ < 20%	10 ± 4	9 ± 4	0.7
	V_18.125 Gy_ < 50%	35 ± 8	37 ± 9	0.7
	V_5 Gy_ (%)	88 ± 12	87 ± 10	0.9
	V_10 Gy_ (%)	67 ± 15	79 ± 10	0.09
	V_20 Gy_ (%)	29 ± 7	28 ± 8	0.8
Rectal wall	D_MAX_ (Gy)	37 ± 2	37 ± 2	0.9
Bowel	V_30 Gy_ < 1 cm^3^	1 ± 2	0.02 ± 0.05	0.1
	D_MAX_ (Gy)	24 ± 10	13 ± 11	0.09
	V_10 Gy_ (%)	4 ± 4	0.2 ± 0.2	0.03
	V_20 Gy_ (%)	0.3 ± 0.5	0.02 ± 0.05	0.1
LFH	V_14.5 Gy_ < 5%	1 ± 2	1 ± 2	0.8
	D_MAX_ (Gy)	15 ± 3	14 ± 2	0.5
RFH	V_14.5 Gy_ < 5%	1 ± 3	0.2 ± 0.5	0.3
	D_MAX_ (Gy)	15 ± 3	13 ± 2	0.3
Penis bulb	V_29.5 Gy_ < 50%	1 ± 3	13 ± 18	0.08
	V_10 Gy_ (%)	28 ± 30	62 ± 30	0.04
	V_20 Gy_ < 90%	8 ± 10	34 ± 30	0.03

**Table 3:** The conformity index, the homogeneity index, the gradient index and the beam on time for the two SBRT methods are reported.

Parameter	CK	VMAT	*p*-Value
CI	1.09 ± 0.04	1.01 ± 0.02	0.0006
HI	1.24 ± 0.03	1.2 ± 0.02	0.01
GI_25_	24 ± 8	22 ± 3	0.01
GI_50_	5 ± 1	5 ± 1	0.6
GI_75_	2.7 ± 0.5	2.5 ± 0.2	0.2
Treatment Time (min)	47 ± 9	3 ± 1	≪0.05

## Data Availability

In this study we do not use raw data that can be used by other researchers. We elaborated treatment plans by using different approaches.
